# Obefazimod in patients with moderate-to-severely active ulcerative colitis: efficacy and safety analysis from the 96-week open-label maintenance phase 2b study

**DOI:** 10.1093/ecco-jcc/jjaf074

**Published:** 2025-05-26

**Authors:** Severine Vermeire, Josianne Nitcheu, Paul Gineste, Aurélien Flatres, Julien Santo, Didier Scherrer, Laurent Peyrin-Biroulet, Parambir S Dulai, Silvio Danese, Marla Dubinsky, Herbert Tilg, Britta Siegmund, Tadakazu Hisamatsu, Kejia Shan, Christopher J Rabbat, Bruce E Sands

**Affiliations:** Department of Gastroenterology and Hepatology, University Hospitals Leuven, Leuven, Belgium; Abivax S.A., Paris, France; Abivax S.A., Paris, France; Abivax S.A., Montpellier, France; Abivax S.A., Montpellier, France; Abivax S.A., Montpellier, France; Department of Gastroenterology, INFINY Institute, INSERM NGERE, CHRU Nancy, Vandœuvre-lès-Nancy, France; Feinberg School of Medicine, Northwestern University, Chicago, IL, United States; Gastroenterology and Endoscopy, IRCCS Ospedale San Raffaele, Milan, Italy; Pediatric GI and Nutrition, Mount Sinai Kravis Children’s Hospital, New York, NY, United States; Department of Gastroenterology, Hepatology, Endocrinology and Metabolism, Medical University of Innsbruck, Innsbruck, Austria; Department of Gastroenterology, Infectiology and Rheumatology, Charité-Universitaetsmedizin Berlin, Corporate Member of Freie Universität Berlin and Humboldt-Universität zu Berlin, Berlin, Germany; Department of Gastroenterology and Hepatology, Kyorin University Hospital, Tokyo, Japan; Abivax S.A., Paris, France; Abivax S.A., Paris, France; Dr. Henry D. Janowitz Division of Gastroenterology, Icahn School of Medicine at Mount Sinai, New York, NY, United States

**Keywords:** obefazimod, miR-124, ulcerative colitis, maintenance

## Abstract

**Background and Aims:**

Obefazimod is an oral small molecule that selectively enhances the expression of a single micro-RNA (miRNA), miR-124. Obefazimod has demonstrated safety and efficacy in patients with moderate-to-severely active ulcerative colitis (UC) in a phase 2b induction trial. This analysis presents the 2-year outcome data of the open-label maintenance (OLM) study.

**Methods:**

Patients received placebo or obefazimod 25, 50, or 100 mg once–daily (od) during the induction trial and, irrespective of their clinical response, could enter the 96-week OLM study with obefazimod 50 mg od. Safety was monitored through monthly visits in the first year and quarterly visits in the second year. Efficacy was assessed at weeks 48 and 96 using nonresponder imputation (NRI) for missing data.

**Results:**

Of 222 eligible patients, 217 were enrolled and 164 (75.6%) completed week 96 of the OLM study. Clinical response was achieved at weeks 48 and 96 in 177 (81.6%) and 158 (72.8%) patients and clinical remission in 119 (54.8%) and 114 (52.5%) of patients. A total of 133 (61.3%) and 128 (59.0%) patients showed endoscopic improvement, and 72 (33.2%) and 78 (35.9%) endoscopic remission. In total, 148/217 patients (68.2%) reported at least 1 treatment-emergent adverse event (TEAE). The most frequent TEAEs were COVID-19 (14.3%), headache (11.5%), UC (7.8%), and nasopharyngitis (6.9%). No new safety risks emerged over 96 weeks.

**Conclusions:**

The 96-week OLM study supports the long-term efficacy and favorable safety profile of obefazimod 50 mg od. A phase 3 program with obefazimod in patients with moderate-to-severe UC is ongoing.

**Trial registration name/number:**

A phase 2b, open-label, efficacy and safety study of ABX464 as maintenance therapy in patients with moderate-to-severe UC. NCT04023396.

## 1. Introduction

Ulcerative colitis (UC) is a chronic inflammatory disease, affecting the colon and rectum, in which patients experience periods of remission and relapse that include symptoms such as bloody diarrhea, urgency, and tenesmus.^1–5^ Patients experience significantly lower health-related quality of life including physical activity, ability to work, and social stigma.^1,2,6–8^ Although the development of advanced targeted therapies has led to better disease control and improvements in patients’ quality of life, most patients fail to achieve clinical remission. Among the minority of patients that do, many will lose efficacy over time, necessitating cycling from 1 treatment to another, with decreasing rates of efficacy. Up to 20% of patients eventually need total colectomy for refractory disease or dysplasia secondary to longstanding uncontrolled inflammation. Most advanced targeted therapies require chronic parenteral administration which can be a burden for some patients. Oral therapies have recently been introduced but come with burdensome pre-initiation assessments (eg, baseline ECG, ocular and skin exams for S1Ps) or carry black box warnings for serious infections, malignancy, and thrombovascular events in their US FDA prescribing information (Janus kinase [JAK] inhibitors). Taken together, these points highlight the unmet need for additional oral therapies with new mechanisms of action that can provide durable efficacy, improved safety, and minimal pre-initiation assessments.

Obefazimod is an investigational, oral, once–daily (od), small molecule that enhances the expression of a single micro-RNA (miR)-124,^9–11^ a natural regulator of the inflammatory response.^12–20^ Evidence from human clinical trials and animal models indicates obefazimod returns multiple cytokines and immune cells to homeostatic levels, helping to stabilize the inflammatory response and potentially modify the progression of UC.^11^ This first-in-class molecule is being investigated as a new treatment option for patients with UC.^11^ A phase 2b randomized, placebo-controlled induction trial evaluated obefazimod in patients with moderately to severely active UC.^21^ More than 45% had failed at least 2 prior advanced targeted therapies (biologics or JAK inhibitors). Obefazimod (25, 50, or 100 mg) significantly reduced the modified Mayo score (MMS) at week 8, meeting the primary endpoint. Improvements were seen in both naïve and bio/JAK refractory patients in key secondary outcomes, including clinical remission, response, endoscopic, and histologic–endoscopic mucosal improvement. Treatment-emergent adverse events (TEAEs), mostly mild-to-moderate, included dose-dependent headaches. At the end of the induction trial, irrespective of their clinical response, patients could enter a 96-week open-label maintenance (OLM) study where they received obefazimod 50 mg od. Here, we report the efficacy and safety findings among the 217 patients who enrolled in the OLM study.

## 2. Methods

### 2.1. Study design and participants

**Figure 1.**
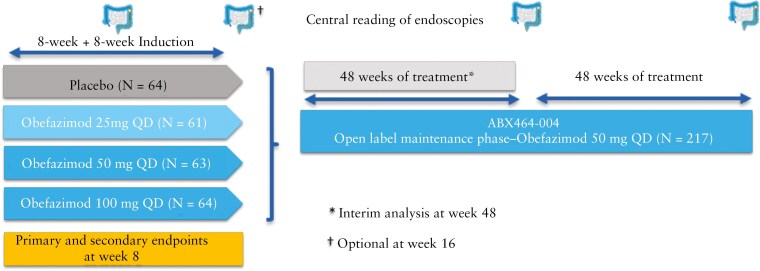
Study design.

The study design and results from the induction trial, as well as the eligibility and exclusion criteria for patients aged 18–75 with moderate-to-severely active UC, have been reported previously.^21^ Of the 252 patients treated in the induction trial, baseline disease activity was severe (MMS of 7–9) in 71.4% of patients, and more than 45% of patients were refractory to 2 or more advanced targeted therapies at baseline. Patients who had completed the induction study phase and who were willing to continue treatment could enter the OLM and receive treatment with obefazimod 50 mg od, regardless of the patient’s clinical response or randomized treatment (placebo or obefazimod 25, 50, or 100 mg od) during induction (Figure 1). The objectives of the OLM study were to assess the long-term safety and efficacy of obefazimod 50 mg od. After week 48, patients who had shown clinical response (defined as a decrease in MMS ≥2 points and ≥30% from baseline (induction), plus a decrease in rectal bleeding subscore [RBS] ≥1 or an absolute RBS ≤1) were eligible for continued participation in the extension for a total period of 96 weeks. From day 1 onwards, patients were seen at the investigational site every 4 weeks up to week 48, then at weeks 60, 72, 84, and 96; flexible sigmoidoscopy with rectal and/or sigmoidal biopsies was performed at weeks 48 and 96 (both central and blinded reading of endoscopies were assessed). Patients were enrolled at 69 study centers in 14 countries (Austria, Belgium, Canada, Czech Republic, France, Germany, Hungary, Italy, Poland, Serbia, Slovakia, Slovenia, Spain, and Ukraine).

Institutional review boards at each study site approved the protocol, and all patients provided written informed consent. The study was undertaken and reported in accordance with the study protocol.

### 2.2. Procedures

Allowed concomitant medications were continued from the baseline of the induction trial. These included corticosteroids (CS; ≤20 mg per day of prednisone or prednisone equivalent, ≤5 mg per day of beclomethasone dipropionate, or ≤9 mg per day budesonide MMX), oral 5-ASA, immunosuppressants such as azathioprine and 6-mercaptopurine, and antidiarrheals. The use of biologics (eg, TNF-α inhibitors, vedolizumab, ustekinumab), JAK inhibitors, cyclosporin, and tacrolimus was prohibited during the study. At the start of the OLM study, CS-tapering was recommended in all patients but was not mandatory.

### 2.3. Outcomes

The efficacy endpoints were evaluated in all patients and in the subgroup of patients that achieved clinical response at week 8 of the induction trial. Endpoints included rate of clinical remission (defined as stool frequency subscore [SFS] ≤1 and rectal bleeding = 0 and endoscopic score of 0 or 1), rate of clinical response (defined as a decrease from baseline in MMS ≥2 points and ≥30% from baseline, plus a decrease in RBS ≥1 or an absolute RBS ≤1), rate of endoscopic improvement (endoscopic subscore ≤1), rate of endoscopic remission (endoscopic subscore = 0), change from baseline of OLM study by visit up to week 96 for stool frequency, and fecal calprotectin (FCP). CS-free status, defined as the cessation of CS for at least 12 weeks prior to each assessment time point in this analysis, was evaluated for efficacy outcomes at weeks 48 and 96 within the subgroup of patients receiving concomitant CS at the induction baseline.

Adverse events were monitored throughout the study. Safety measures comprised the incidence of TEAEs, serious treatment-emergent adverse events (TESAEs), discontinuations due to TEAEs, drug-related SAEs, and clinically significant laboratory abnormalities.

Absolute quantification of the miR-124 copy number in rectal biopsies and blood samples was performed at baseline, weeks 48 and 96 by using droplet digital PCR (ddPCR) technology on 115 whole blood samples and 240 rectal biopsy samples (see Supplementary data for full details on methods of measurement).

### 2.4. Statistical analysis

The primary analysis set for efficacy was the full analysis set (FAS, *N* = 217), and analysis of safety parameters was carried out using the safety analysis set (SAF, *N* = 217). The FAS included all patients who received at least 1 dose of obefazimod 50 mg and had a baseline of OLM data for at least 1 efficacy variable; the SAF included all patients who received at least 1 dose of obefazimod 50 mg.

Baseline of OLM was defined as the last non-missing measurement after induction (week 8 or 16) taken prior to the first dose of obefazimod 50 mg of the OLM study.

Descriptive statistics were presented for all efficacy and safety variables. The proportions of patients in clinical remission, clinical response, with endoscopic improvement, or endoscopic remission were summarized descriptively using nonresponder imputation (NRI) analysis at weeks 48 and 96 (patients with missing data were imputed as nonresponders). Descriptive subgroup analyses were conducted based on patients’ status (clinical response yes/no) at week 8 of the induction trial, naïve or had prior inadequate response to biologics/JAK inhibitors (defined as nonresponse, loss of response, or intolerance), and corticosteroid status.

The enhanced expression of miR-124 was quantified as a fold change relative to baseline. An ANOVA with Dunnett’s multiple comparisons tests was performed including treatment, time, the interaction between treatment and time, the baseline value as a fixed factor, and time as repeated effect. The *P*-value threshold for statistically significant differences was *P* < .05. SAS version 9.4 (SAS Institute, Inc.) was used for all statistical analyses.

## 3. Results

### 3.1. Patient disposition and baseline characteristics

Of the 222 eligible patients who completed the induction trial, 217 (97.7%) were enrolled in the OLM study (Figure S1). At baseline of OLM, the mean age was 42.1 years; 61.3% of patients were men; 162 patients were randomized to 1–3 doses of obefazimod and 55 patients to placebo in the induction trial. At baseline of OLM, 98 patients (45.2%) had prior inadequate response to biologics or JAK inhibitors (70 of these 98 (71.4%) patients had inadequate response to ≥2 biologics or JAK inhibitors), 119/217 patients (54.8%) were naive to biologics or JAK inhibitors and 120/217 patients (55.3%) were using concomitant corticosteroids (Table 1). Thirty of 217 (13.8%) discontinued prior to week 48, 6 patients did not meet eligibility criteria for the second year of treatment (did not achieve clinical response at week 48 or were unwilling to continue) and 17/181 (9.4%) patients discontinued between weeks 48 and 96. All discontinuations were considered as treatment failures for the NRI analysis of efficacy at weeks 48 and 96.

**Table 1.** Demographic and baseline characteristics.

		Total (*N* = 217)
Age (years)	Mean (SD)	42.1 (13.8)
Male	*n* (%)	133 (61.3)
Female	*n* (%)	84 (38.7)
Treatment received during the induction trial		
Obefazimod 25 mg	*n* (%)	58 (26.7)
Obefazimod 50 mg	*n* (%)	51 (23.5)
Obefazimod 100 mg	*n* (%)	53 (24.4)
Placebo	*n* (%)	55 (25.3)
Naïve to Biologics/JAK inhibitors	*n* (%)	119 (54.8)
Inadequate response to Biologics/JAK inhibitors Inadequate response to ≥2 Biologics/JAK inhibitors	*n*1 (%) *n*2 (%^a^)	98 (45.2) 70 (71.4)
Patients using concomitant corticosteroids	*n* (%)	120 (55.3)
Patients using concomitant 5-ASA	*n* (%)	159 (73.3)
Fecal calprotectin (µg/g)	Mean (SD)	900.1 (1574.5)
	Median [range]	204.7 [14.2–10 405.9]

Abbreviations: 5-ASA, 5-aminosalicylic acid; MMS, modified Mayo score; *n*, number of patients in the relevant category; *N*, number of patients in the relevant analysis set; SD, standard deviation.

Percentages are calculated relevant to the number of patients in the relevant analysis set.

^a^ *n*2/*n*1 × 100. Baseline refers to the baseline of open-label maintenance (OLM).

### 3.2. Efficacy

**Figure 2.**
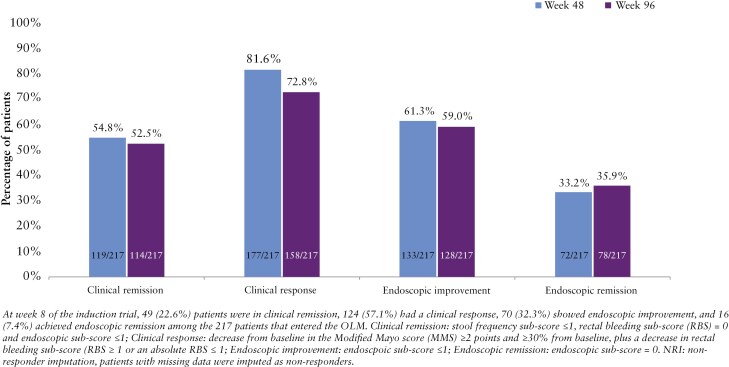
Efficacy at weeks 48 and 96 in all patients (NRI).

Among the 217 patients who enrolled in the OLM study and received 50 mg od oral dosing with obefazimod, 177/217 (81.6%) and 158/217 (72.8%) achieved clinical response at weeks 48 and 96, respectively, and 119/217 (54.8%) and 114/217 (52.5%) patients achieved clinical remission at weeks 48 and 96, respectively. Furthermore, 133/217 (61.3%) and 128/217 (59.0%) patients had endoscopic improvement at weeks 48 and 96, while 72/217 (33.2%) and 78/217 (35.9%) patients attained endoscopic remission at weeks 48 and 96, respectively (Figure 2). The mean SFS decreased from 1.4 at OLM baseline to 0.7 at week 48 and 0.6 at week 96, with most patients having an SFS of 0 or 1 (87.9%) at week 96.

The median FCP concentration decreased from 204.7 µg/g at baseline of OLM, to 86.6 µg/g at week 48 and 89.6 µg/g at week 96 (Table 2). Of the 167 patients with available data at week 48, 122/167 (73.1%) achieved FCP levels below 250 µg/g, and 109/167 (65.3%) achieved FCP levels below 150 µg/g. At week 96, 102 (69.4%) of the 147 patients with available data achieved FCP levels below 250 µg/g, and 91/147 (61.9%) achieved FCP levels below 150 µg/g.

**Table 2.** Fecal calprotectin (data as observed).

Fecal calprotectin (µg/g)		Total (*N* = 217)
Baseline of OLM	*n*	192
	Median [range]	204.7 [14.2–10 405.9]
<150 µg/g	*n* (%)	82 (42.7)
<250 µg/g	*n* (%)	101 (52.6)
Week 48	*n*	167
	Median [range]	86.6 [14.2–6101.4]
<150 µg/g	n (%)	109 (65.3)
<250 µg/g	n (%)	122 (73.1)
Patients with reduction relative to baseline of OLM	*n* (%)	97 (58.1)
Week 96	*n*	147
	Median [range]	89.6 [14.2–7346.1]
<150 µg/g	*n* (%)	91 (61.9)
<250 µg/g	*n* (%)	102 (69.4)
Patients with reduction relative to baseline of OLM	*n* (%)	84 (57.1)

*n*, Number of patients in the relevant category; *N*, number of patients in the relevant analysis set; OLM, open-label maintenance; SD, standard deviation.

### 3.3. Efficacy in clinical responders and nonresponders at week 8 of induction trial

**Figure 3.**
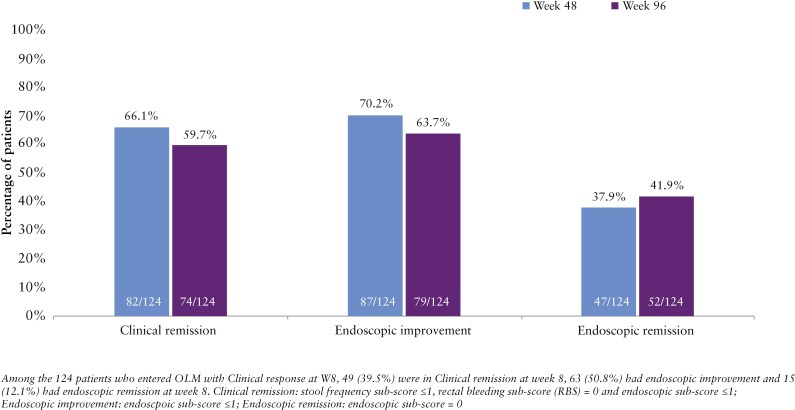
Efficacy at weeks 48 and 96 in clinical responders at week 8 of the induction study.

Among the 124 patients with clinical response at week 8 of the induction trial, 82/124 (66.1%) and 74/124 (59.7%) achieved clinical remission at weeks 48 and 96, respectively (Figure 3). Additionally, 87/124 (70.2%) and 79/124 (63.7%) achieved endoscopic improvement, while 47/124 (37.9%) and 52/124 (41.9%) achieved endoscopic remission at weeks 48 and 96, respectively. Among the 124 patients that achieved clinical response at week 8, 79/124 (63.7%) were naïve to biologics or JAK inhibitors, while 45/124 (36.3%) had inadequate response to 1 or more of these therapies.

Among the 93 patients that did not achieve clinical response at week 8 of the induction trial, 37/93 (39.8%) and 40/93 (43.0%) achieved clinical remission at weeks 48 and 96, respectively. Additionally, 46/93 (49.5%) and 49/93 (52.7%) had endoscopic improvement, 25/93 (26.9%) and 26/93 (28.0%) achieved endoscopic remission at weeks 48 and 96, respectively. Among these patients, 40/93 (43.0%) were biologic or JAK inhibitor-naïve, and 53/93 (57.0%) had a prior inadequate response to 1 or more of these therapies.

### 3.4. Efficacy stratified by whether patients received placebo or obefazimod during induction

Overall, 162 patients treated with obefazimod during induction entered the OLM. Among this subgroup, 103/162 (63.6%) achieved clinical response at W8 of the induction trial, while 59/162 (36.4%) did not. At both W48 and W96, the proportion of patients achieving efficacy endpoints was numerically higher among W8 clinical responders than among W8 nonresponders (Table S1). For example, clinical remission was achieved by 69/103 (67.0%) and 65/103 (63.1%) among W8 clinical responders at W48 and W96, respectively, compared to 20/59 (33.9%) and 24/59 (40.7%) among W8 nonresponders.

Among the 55 patients receiving placebo during induction and entering the OLM, 21/55 (38.2%) achieved clinical response at W8 of the induction trial, while 34/55 (61.8%) did not. Clinical remission was achieved by 13/21 (61.9%) and 9/21 (42.9%) of W8 clinical responders at W48 and W96, respectively, compared to 17/34 (50.0%) and 16/34 (47.1%) of nonresponders.

### 3.5. Subsets of patients naïve or with inadequate response to biologics or JAK inhibitors

**Figure 4.**
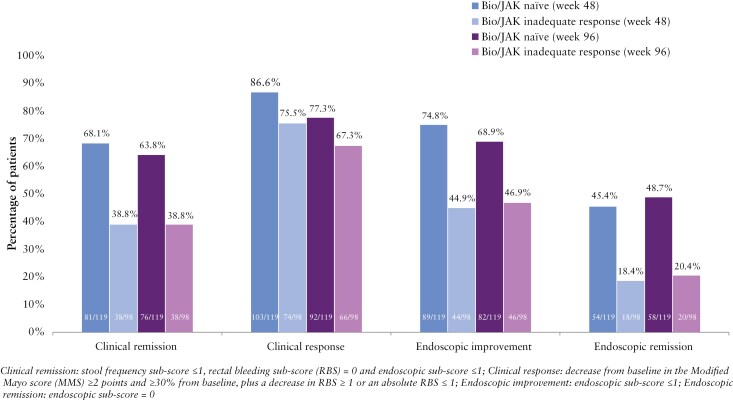
Efficacy at week 48 and week 96 in patients with inadequate response to biologics or Janus kinase (JAK) inhibitors or naïve to biologics or JAK inhibitors at induction baseline.

Among the 98 patients with inadequate response to biologics (anti-TNFα, vedolizumab, ustekinumab) or JAK inhibitors, 74/98 (75.5%) achieved clinical response at week 48 and 66/98 (67.3%) at week 96; 38/98 patients (38.8%) were in clinical remission at both weeks 48 and 96 (Figure 4). Endoscopic improvement was observed in 44/98 (44.9%) and 46/98 (46.9%) patients at weeks 48 and 96, while endoscopic remission was achieved by 18/98 (18.4%) and 20/98 (20.4%) patients at weeks 48 and 96, respectively. Among the 119 patients naïve to biologics or JAK inhibitors at the induction study baseline, 103/119 (86.6%) and 92/119 (77.3%) achieved a clinical response at weeks 48 and 96. In this subset, clinical remission was achieved by 81/119 patients (68.1%) at week 48 and by 76/119 patients (63.8%) at week 96; 89/119 (74.8%) and 82/119 patients (68.9%) had endoscopic improvement, and 54/119 (45.4%) and 58/119 patients (48.7%) achieved endoscopic remission at weeks 48 and 96, respectively.

### 3.6. Subsets of patients receiving concomitant CS at induction baseline

**Figure 5.**
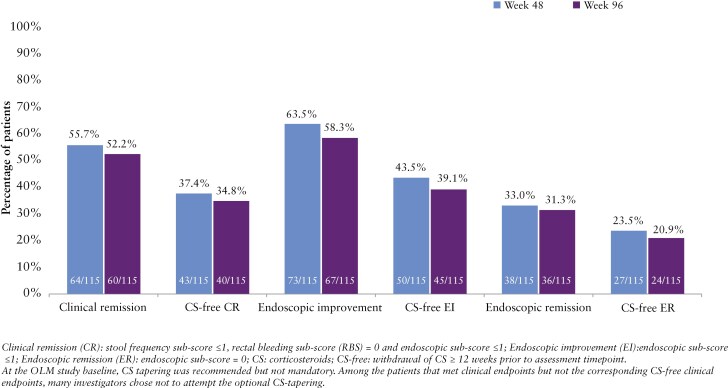
Efficacy and CS-free efficacy at weeks 48 and 96 in patients with concomitant CS at induction baseline.

Among the 115 patients enrolled in the OLM who received concomitant CS at induction baseline, 64/115 (55.7%) were in clinical remission rates at week 48 and 60/115 (52.2%) at week 96. While the protocol did not require CS-tapering upon entry to the OLM study, 12-week CS-free clinical remission was attained by 43/115 patients (37.4%) at week 48 and by 40/115 patients (34.8%) at week 96. Endoscopic improvement was observed in 73/115 patients (63.5%) at week 48 and in 67/115 patients (58.3%) at week 96. CS-free endoscopic improvement was achieved by 50/115 patients (43.5%) at week 48 and by 45/115 patients (39.1%) at week 96. Endoscopic remission was achieved by 38/115 patients (33.0%) at week 48 and by 36/115 patients (31.3%) at week 96, with CS-free endoscopic remission achieved by 27/115 patients (23.5%) at week 48 and 24/115 patients (20.9%) at week 96 (Figure 5). Notably, among the patients that met clinical endpoints but not the corresponding CS-free clinical endpoints, many investigators chose not to attempt the optional CS-tapering.

### 3.7. miR-124 modulation in rectal tissue and blood samples

**Figure 6.**
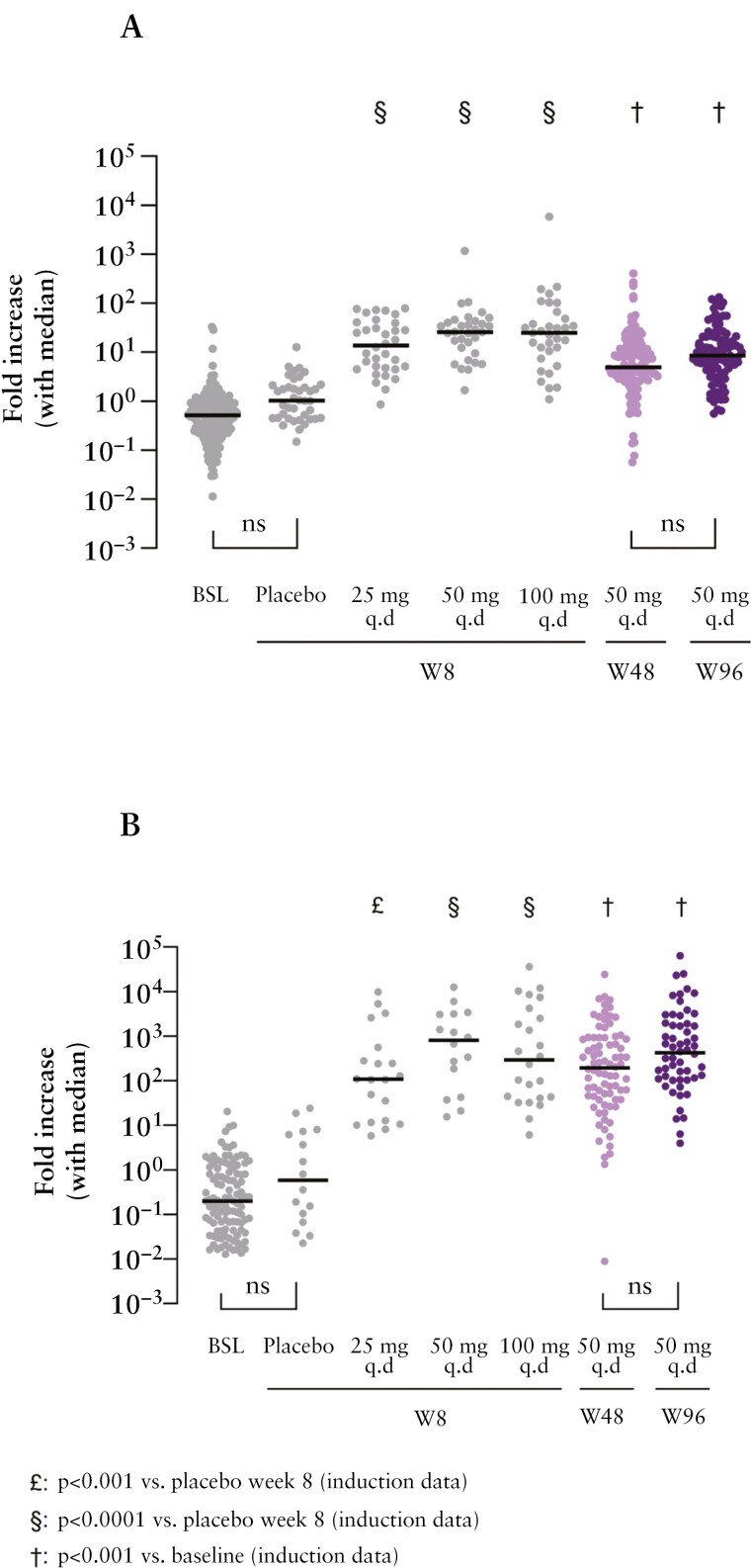
MiR-124-enhanced expression in rectal tissue (A) and blood (B) in ulcerative colitis (UC) patients. The number of miR-124 copies was assessed by droplet digital PCR (ddPCR) at baseline (BSL), weeks 8, 48, and 96.

Treatment with obefazimod 50 mg significantly enhanced the expression of miR-124 in blood and rectal tissue at weeks 48 and 96 (*P* < .001 vs. baseline) (Figure 6). In the blood and rectal tissue of patients, the median fold change from baseline was numerically higher at week 96 compared to week 48. Enhanced expression of miR-124 was observed in both rectal tissue and blood of patients receiving obefazimod 50 mg during the OLM study, including those previously receiving placebo during the induction study (Table S2).

### 3.8. Safety

At least 1 TEAE was reported for 148 (68.2%) of 217 obefazimod-treated patients (Table 3). The most common TEAEs reported in at least 5% of patients were COVID-19 (14.3%), headache (11.5%), UC (7.8%), nasopharyngitis (6.9%), back pain (5.5%), and arthralgia (5.1%). TESAEs were reported by 18 patients (8.3%). Proctitis and COVID-19 were the most commonly reported TESAEs (2 patients, 0.9%); other TESAEs occurred in no more than one patient (Table S3). Infections and infestations TESAEs, reported for 4 patients, comprised COVID-19/COVID-19 pneumonia (2 patients, 1.0%), urosepsis (1 patient, 0.5%), and appendicitis (1 patient, 0.5%). There was 1 malignant meningioma unrelated to treatment, and 1 death was reported, unrelated to treatment (car accident). TEAEs leading to study discontinuation were reported in 17 patients (7.8%) (Table 4). These events included the worsening of disease (coded as colitis ulcerative and colitis), which resulted in the discontinuation of 9 patients. Twenty-two patients (10.1%) had 30 treatment-emergent AESIs. AESIs reported in more than 1 patient were headache, rash, and eczema, which were reported in 4 (1.8%), 3 (1.4%), and 2 patients (0.9%), respectively. Laboratory results revealed no new or unexpected safety signals, and there were no clinically meaningful changes in laboratory parameters compared to week 8.

**Table 3.** Treatment-emergent adverse events occurring in ≥3% of patients.

Any TEAE, *n*	148 (68.2
COVID-19	31 (14.3)
Headache	25 (11.5)^a^
Colitis ulcerative	17 (7.8)
Nasopharyngitis	15 (6.9)
Back pain	12 (5.5)
Arthralgia	11 (5.1)
Fecal calprotectin increased	10 (4.6)
Nausea	8 (3.7)
Abdominal pain	8 (3.7)
Blood cholesterol increased	8 (3.7)
Hypertension	8 (3.7)
Hemorrhoids	7 (3.2)

Abbreviations: *n*, number of patients in the relevant category; OLM, open-label maintenance; TEAE, treatment-emergent adverse event.

^a^Ten of the 25 patients who reported headache experienced them within the first 7 days after switching from placebo to obefazimod or from 25 to 50 mg upon entering the OLM study. Percentages are calculated relevant to the number of patients in the safety set (*N* = 217).

**Table 4.** Treatment-emergent adverse events leading to study discontinuation.

Any TEAE, *n* (%)	17 (7.8)
Colitis ulcerative	7 (3.2)
Colitis	2 (0.9)
Nausea	1 (0.5)
Headache	1 (0.5)
Ischemic stroke	1 (0.5)
Polyneuropathy	1 (0.5)
Left ventricular dysfunction	1 (0.5)
Injury^a^	1 (0.5)
Alanine aminotransferase increased	1 (0.5)
Joint effusion	1 (0.5)

Abbreviations: TEAE, treatment-emergent adverse event.

Percentages are calculated relevant to the number of patients in the safety set (*N* = 217).

^a^Car accident leading to death, unrelated to treatment.

## 4. Discussion

In patients with moderate-to-severely active UC, obefazimod 50 mg QD was effective in improving patients’ condition at weeks 48 and 96 of this open-label study, based on clinical, endoscopic, and biomarker measures. The patient retention rate over 2 years was high (164/217 patients, 75.6%), and the long-term efficacy findings using NRI analysis were robust and remarkably consistent at week 96 relative to week 48. Specifically, at week 96, 52.5% of patients achieved clinical remission, closely matching the rate at week 48 (54.8%). Similarly, endoscopic improvement was observed in 59.0% of patients at week 96 compared to 61.3% at week 48. Endoscopically determined improvement in mucosal appearance has been associated with more favorable long-term outcomes in patients with UC.^22^ Biomarkers data supported the clinical findings with fecal calprotectin (FCP)^23,24^ decreasing at weeks 48 and 96 relative to baseline of this OLM study. At week 48, FCP concentration was below 150 µg/g for 65% of patients, and 62% of patients at week 96. FCP levels below 150 µg/g typically indicate remission with a high degree of accuracy according to Selecting Therapeutic Targets in Inflammatory Bowel Disease (STRIDE-II) recommendations.^25^ These data suggest that the long-term improvements with obefazimod are mediated through reducing the pro-inflammatory response.

Consistent with other advanced targeted therapies, efficacy outcomes at weeks 48 and 96 in the subgroup of 98 patients who had inadequate response to bio/JAK inhibitor treatment were lower relative to the population naïve to bio/JAK inhibitor treatment. These patients were particularly refractory, as more than 70% of them had inadequate response to at least 2 biologics or JAK inhibitors. Rates of clinical remission, endoscopic improvement, and endoscopic remission at weeks 48 and 96 were numerically higher, from 25% to 33%, in the subgroup of naïve relative to refractory patients. The notable difference in endoscopic remission rates between bio/JAK-naïve patients (45.4% at week 48 and 48.7% at week 96) and refractory patients (18.4% at week 48 and 20.4% at week 96) may be due to the cumulative impact of chronic inflammation in refractory individuals. Chronic inflammation can lead to structural damage to the colon, such as scarring and fibrosis, which makes full endoscopic remission more difficult to achieve, even when inflammation is controlled.^26^

Among patients receiving concomitant CS at induction baseline, meaningful proportions achieved clinical remission, endoscopic improvement, and endoscopic remission at both weeks 48 and 96 in the OLM study, with proportions similar to those observed in the overall population. Notably, a meaningful proportion of patients met CS-free efficacy endpoints, even though CS-tapering was not mandatory in this study and many investigators chose not to taper in patients who achieved clinical endpoints. CS-free efficacy rates might have been higher had CS-tapering been required during the OLM study.

Efficacy rates increased during the maintenance treatment period with obefazimod 50 mg relative to week 8 of the induction trial. The percentage of patients achieving clinical remission at week 8 of the induction trial ranged from 18% to 26% in the obefazimod groups,^21^ while 55% did so at week 48 of the OLM study. Similarly, at week 8 of the induction trial, the percentage of patients achieving clinical response and endoscopic improvement ranged from 50% to -62% and 33% to 38%, respectively. At week 48 of the OLM study, these percentages increased to 82% for clinical response and 61% for endoscopic improvement. The longer time required to observe clinical and endoscopic benefits likely reflects ongoing healing with long-term treatment.

The long-term efficacy of obefazimod 50 mg QD in patients with moderate-to-severe UC is associated with a sustained increase in miR-124 expression in blood and rectal tissue. The sustained enhanced expression of miR-124 for 2 years may be associated with the durability of clinical response observed with obefazimod in this study.

The main adverse events during the OLM study were headache, COVID-19, colitis ulcerative, nasopharyngitis, back pain, and arthralgia. Headache appears to be associated with the introduction to obefazimod treatment, as the rate was reduced to 11.5% of patients who received up to 96 weeks obefazimod 50 mg compared to 30.2% of patients who received obefazimod 50 mg during induction.^21^ UC (ie, worsening of UC) was the most frequently reported adverse event that led to discontinuation during the study. Only 1 malignancy was reported and judged not related to treatment, and appendicitis, urosepsis, and COVID-19 were the only serious infectious adverse events. This study revealed no clinically meaningful changes in laboratory parameters throughout the study.

In conclusion, this study supports the long-term efficacy and safety of obefazimod 50 mg QD, with a substantial proportion of patients achieving clinical remission at weeks 48 and 96. Consistent improvement in endoscopy scores and FCP was observed over time. These findings suggest that long-term treatment with obefazimod provides continued improvement of clinical symptoms of the disease. Safety data were consistent with the established safety profile of obefazimod. Although the central evaluation of endoscopy data reduced the risk of bias in this open-label study, the results must be interpreted with caution due to the absence of a control group. Furthermore, steroid cycling was not accounted for in the NRI method, potentially limiting the interpretation of CS-free outcomes. The safety and efficacy of obefazimod are being further assessed in the ongoing double-blind, randomized, placebo-controlled phase 3 ABTECT program (NCT05507203, NCT05507216, NCT05535946).

## Supplementary Material

jjaf074_suppl_Supplementary_Figure_S1_Tables_S1-S3

## Data Availability

The data underlying this article will be shared on reasonable request to the corresponding author.
