# Supplementary material for: Obefazimod in patients with moderate-to-severely active ulcerative colitis: efficacy and safety analysis from the 96-week open-label maintenance phase 2b study
Source: J Crohns Colitis. 2025 May 26;19(5):jjaf074. doi: 10.1093/ecco-jcc/jjaf074 (PMC12124117; doi:10.1093/ecco-jcc/jjaf074)
Supplement: jjaf074_suppl_Supplementary_Figure_S1_Tables_S1-S3 [file jjaf074_suppl_supplementary_figure_s1_tables_s1-s3.docx]

**Supplementary data**:

*Quantification of miR-124*

Absolute quantification of the miR-124 copy number in rectal biopsies and blood samples was performed at baseline, weeks 48 and 96 by using droplet digital PCR (ddPCR) technology on 115 whole blood samples and 240 rectal biopsy samples. RNA and DNA were co-extracted with the quick-DNA/RNA Magbead kit (Zymo) and quantified using the Qubit 2.0 fluorometer and the Qubit RNA (or DNA) High Sensitivity Kit (ThermoFisher). The miRNAs were retrotranscribed using the TaqMan Advanced miRNA cDNA RNA Synthesis Kit (ThermoFisher), followed by preamplification to ensure that miR-124 and miR16 were present. The ddPCR was performed on a QX200 droplet digital PCR system, and the data were analyzed with QuantaSoft Pro v1.7.4.0917. The preamplified cDNA was subjected to duplicate ddPCR assays measurements using two singleplex assays targeting miR-124 and miR-16 (a housekeeping gene for miR-124 normalization). For each sample, miR-124 and miR-16 were measured on the same ddPCR plate in a singleplex reaction and were analyzed together. The total copy number *per* sample was calculated by adjusting for all dilution factors. The normalized miR16 copy number was calculated using a sample-specific normalization factor that considers the relative expression of miR-16 *per* sample *per* batch.

**Supplementary Table 1**: Efficacy at weeks 48 and 96 of OLM study among clinical responders and non-responders at week 8 of induction trial

|  | **Patients treated with obefazimod during induction (N=162)** | | | | **Patients receiving placebo during induction (N=55)** | | | |
| --- | --- | --- | --- | --- | --- | --- | --- | --- |
|  | **Clinical response at week 8 (N=103)** | | **No clinical response at week 8 (N=59)** | | **Clinical response at week 8 (N=21)** | | **No clinical response at week 8 (N=34)** | |
| **Efficacy Endpoint, % (n)** | **Week 48** | **Week 96** | **Week 48** | **Week 96** | **Week 48** | **Week 96** | **Week 48** | **Week 96** |
| **Clinical remission** | 67.0 (69) | 63.1 (65) | 33.9 (20) | 40.7 (24) | 61.9 (13) | 42.9 (9) | 50.0 (17) | 47.1 (16) |
| **Clinical response** | 87.4 (90) | 80.6 (83) | 69.5 (41) | 57.6 (34) | 81.0 (17) | 57.1 (12) | 88.2 (30) | 85.3 (29) |
| **Endoscopic improvement** | 71.8 (74) | 68.0 (70) | 40.7 (24) | 49.2 (29) | 61.9 (13) | 42.9 (9) | 64.7 (22) | 58.8 (20) |
| **Endoscopic remission** | 35.9 (37) | 45.6 (47) | 22.0 (13) | 28.8 (17) | 47.6 (10) | 23.8 (5) | 35.3 (12) | 26.5 (9) |
| Non-responder imputation (NRI) was used for missing efficacy data; clinical remission: stool frequency sub-score ≤1, RBS = 0 and endoscopic sub-score ≤1; clinical response: decrease from baseline in the Modified Mayo score (MMS) ≥ 2 points and ≥30% from baseline, plus a decrease in rectal bleeding sub-score (RBS) ≥ 1 or an absolute RBS ≤ 1; endoscopic improvement: endoscopic sub-score ≤1; endoscopic remission: endoscopic sub-score = 0 | | | | | | | | |

**Supplementary Table 2**: Enhanced expression of miR-124 in rectal tissue and blood of patients receiving obefazimod 50 mg during the OLM study

| **Treatment during the induction study** | **Time** | **Rectal tissue**  Median fold change from induction baseline | **Blood**  Median fold change from induction baseline |
| --- | --- | --- | --- |
|  |  |  |  |
| Obefazimod 100 mg od | Week 48 | 4.8 ^†^ | 128.2 ^†^ |
|  | Week 96 | 7.4 ^†^ | 188.3 ^†^ |
| Obefazimod 50 mg od | Week 48 | 5.8 ^†^ | 230.1 ^†^ |
|  | Week 96 | 10.3 ^†^ | 244.4 ^†^ |
| Obefazimod 25 mg od | Week 48 | 4.5 ^†^ | 308.8 ^†^ |
|  | Week 96 | 8.5 ^†^ | 128.5 ^†^ |
| Placebo | Week 48 | 4.8 ^†^ | 237.0 ^†^ |
|  | Week 96 | 7.5 ^†^ | 396.8 ^†^ |

*†: p<0.001 vs. baseline; each timepoint (week 48 and week 96) is compared to the induction baseline using a Dunnett adjustment.*

**Supplementary Table 3**: Treatment-Emergent Serious Adverse Events - Safety Analysis Set

|  |  |
| --- | --- |
|  |  |
| Any TESAE n (%) | 18 (8.3) |
| Proctitis | 2 (0.9) |
| Colon dysplasia | 1 (0.5) |
| Vomiting | 1 (0.5) |
| Appendicitis | 1 (0.5) |
| COVID-19 | 1 (0.5) |
| COVID-19 pneumonia | 1 (0.5) |
| Urosepsis | 1 (0.5) |
| Facial paresis | 1 (0.5) |
| Ischaemic stroke | 1 (0.5) |
| Polyneuropathy | 1 (0.5) |
| Vertebrobasilar insufficiency | 1 (0.5) |
| Left ventricular dysfunction | 1 (0.5) |
| Palpitations | 1 (0.5) |
| Injury* | 1 (0.5) |
| Meningioma malignant | 1 (0.5) |
| Depression | 1 (0.5) |
| Ureterolithiasis | 1 (0.5) |
| Cervical dysplasia | 1 (0.5) |
| Percentages are calculated relevant to the number of patients in the safety set (N=217). TESAE = Treatment Emergent Serious Adverse Event.  *Car accident leading to death, unrelated to treatment | |

**Supplementary figure 1:** Consort flow diagram

Obefazimod 100 mg (N=64)

Obefazimod 25 mg

(N=63)

Obefazimod 50 mg

(N=63)

**Treatment assignment in induction study**

Placebo

(N=64)

**Treatment assignment at induction phase**

Placebo

(n=64)

Excluded (n = 5)

Allocated to obefazimod 50 mg od

(n = 217)

Assessed for eligibility into OLM

(n = 222)

53 patients discontinued OLM

- - Adverse event (n = 16)
  - Death (n = 1)
  - Lost to Follow Up (n = 1)
  - Pregnancy (n = 4)
  - Progressive Disease (n = 3)
  - Physician Decision (n = 4)
  - Patient Decision (n = 14)
  - Other (n = 10)

-

(give reasons)

Allocated to obefazimod 50 mg od

(n = 217)

Assessed for eligibility

(n = 222)

Lost to follow up (n = 53)

- - Adverse event (n = 16)
  - Death (n = 1)
  - Lost to Follow Up (n = 1)
  - Pregnancy (n = 4)
  - Progressive Disease (n = 3)
  - Physician Decision (n = 4)
  - Subject Decision (n = 9)
  - Other (e.g., Ukraine Crisis) (n = 15)

-

(give reasons)

**Treatment assignment in OLM study**

32 patients discontinued treatment before week 16

164 patients

completed the two years of treatment.

**Treatment assignment at maintenance phase**

32 patients discontinued treatment before week 16

164 patients

completed the two years of treatment.
